# Strongly Bent Double-Stranded DNA: Reconciling Theory and Experiment

**DOI:** 10.3389/fphy.2019.00195

**Published:** 2019-11-29

**Authors:** Aleksander V. Drozdetski, Abhishek Mukhopadhyay, Alexey V. Onufriev

**Affiliations:** 1 Department of Physics, Virginia Tech, Blacksburg, VA, United States,; 2 Department of Computer Science, Virginia Tech, Blacksburg, VA, United States,; 3 Center for Soft Matter and Biological Physics, Virginia Tech, Blacksburg, VA, United States

**Keywords:** polymer bending, DNA, convex hull, deformation, cyclization, *j-factor*

## Abstract

The strong bending of polymers is poorly understood. We propose a general quantitative framework of polymer bending that includes both the weak and strong bending regimes on the same footing, based on a single general physical principle. As the bending deformation increases beyond a certain (polymer-specific) point, the change in the convexity properties of the effective bending energy of the polymer makes the harmonic deformation energetically unfavorable: in this strong bending regime the energy of the polymer varies linearly with the average bending angle as the system follows the convex hull of the deformation energy function. For double-stranded DNA, the effective bending deformation energy becomes non-convex for bends greater than ~ 2° per base-pair, equivalent to the curvature of a closed circular loop of ~ 160 base pairs. A simple equation is derived for the polymer loop energy that covers both the weak and strong bending regimes. The theory shows quantitative agreement with recent DNA cyclization experiments on short DNA fragments, while maintaining the expected agreement with experiment in the weak bending regime. Counter-intuitively, cyclization probability (*j-factor*) of very short DNA loops is predicted to increase with decreasing loop length; the *j-factor* reaches its minimum for loops of ≃ 45 base pairs. Atomistic simulations reveal that the attractive component of the short-range Lennard-Jones interaction between the backbone atoms can explain the underlying non-convexity of the DNA effective bending energy, leading to the linear bending regime. Applicability of the theory to protein-DNA complexes, including the nucleosome, is discussed.

## 1. INTRODUCTION

Deformation of polymers is ubiquitous, elastic properties of these macromolecules are crucial for their dynamics. Biopolymers are abundant in nature and play vital roles in many biological processes [1–4], which not only depend upon the polymer structure, but also on its physical properties [5–7]. Among biopolymers, DNA stands out as a case of its own. Understanding DNA deformation is crucial for the mechanistic grasp of vital cellular functions, such as packaging of DNA compactly into viral capsids, chromatin compaction, formation of protein/DNA complexes and regulation of gene expression [2, 8]. An all-important example of DNA deformation, relevant to a variety of biological processes that depend on its elastic properties, is DNA looping, which occurs in many prokaryotic [9] and eukaryotic [10] systems. A number of regulatory proteins can loop DNA into various bent conformations, critical for regulation of many biological processes involving DNA [11]. Most notably, DNA is strongly bent in the nucleosome [12, 13], which is the fundamental unit of genome packing: accessibility to genomic information in eukaryotes is modulated by the strength of DNA-protein association [14, 15]. Note that the majority of eukaryotic genomic DNA (75–80%) is packed tightly into nucleosomes [10]. Nanostructures made directly of DNA [16] or those that use DNA as a scaffold [17], can be influenced [18] by its mechanical properties on short length scales, providing yet another impetus to understand the strong bending regime of the DNA. Experimental evidence on cyclization of DNA fragments shorter than ~100 base-pairs points to the fact that strongly bent DNA—most relevant from a biological perspective—is considerably more flexible than expected from established models (worm-like chain) that work well within the weak bending regime. Yet, despite decades of experimental and theoretical effort, the story of how this very important polymer behaves under deformation is far from complete, with new developments abound [19–35]. A snapshot of the current state and the relevant terminology are briefly reviewed below.

Bending flexibility of a polymer is conventionally quantified in terms of its persistence length, *L*_*p*_, a length scale below which the polymer behaves more or less like a rigid rod. Specifically, *L*_*p*_ is defined as length of the polymer segment over which the time-averaged orientation of the polymer becomes uncorrelated; for fragments smaller than *L*_*p*_, the thermal fluctuation alone is not enough to induce significant (~ 1 rad) bending [36]. Here we use this definition of *L*_*p*_ to qualitatively separate the two bending regimes: if no significant bending is observed on length scales shorter than *L*_*p*_, the polymer can be deemed *weakly* bent; otherwise the bending is assumed to be *strong*. For double-stranded DNA, a variety of experimental techniques [37–42], revealed that *L*_*p*_ ≈ 150 bp or 500 Å. Based on the *L*_*p*_ value and the above definition of strong bending, we conclude that most of the DNA in eukaryotes is strongly bent. Indeed, since the nucleosome contains a stretch of double-stranded DNA of ~ 150 bp looped almost twice, the DNA in this complex can be considered as strongly bent.

**FIGURE 1 |:**
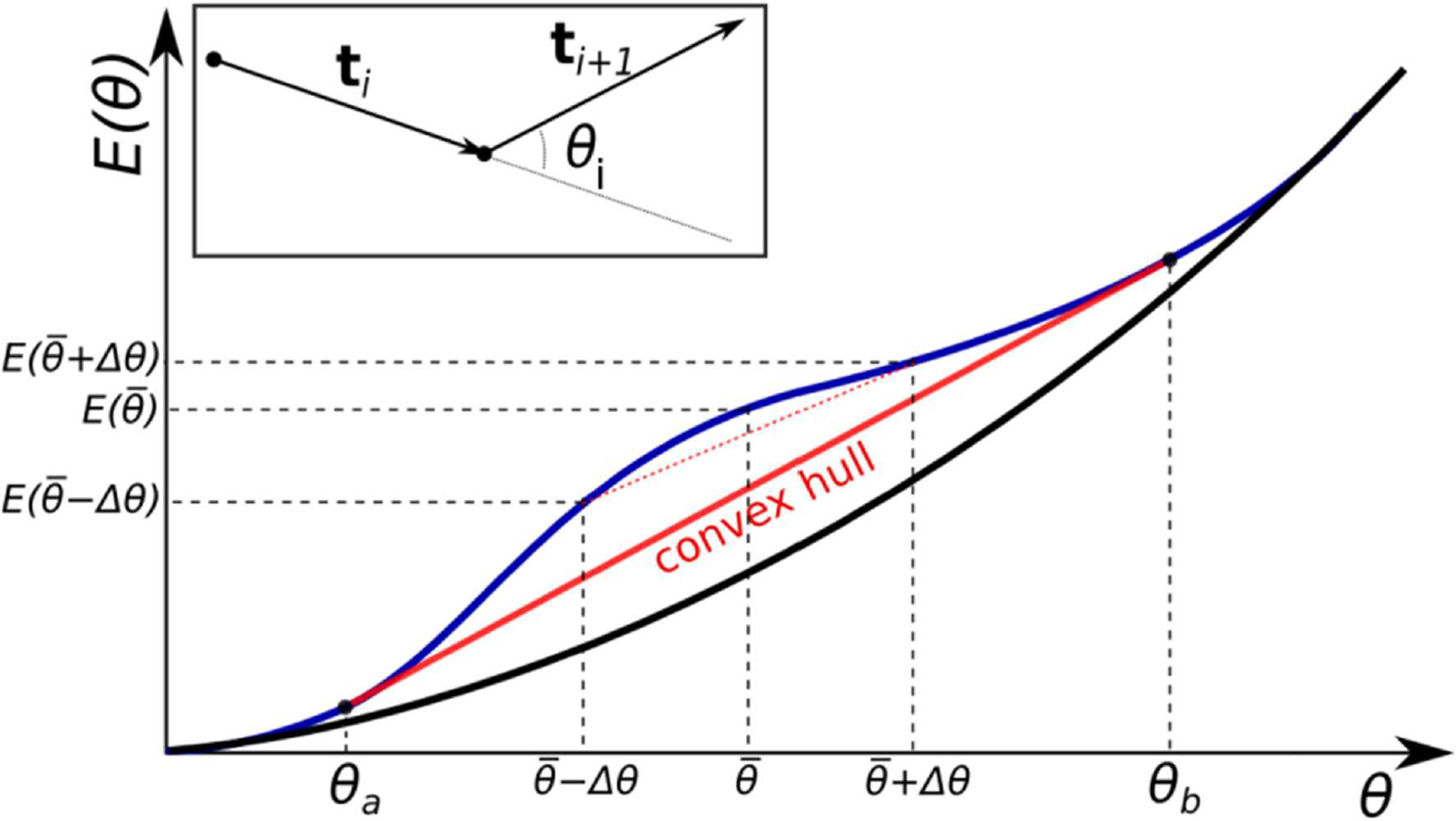
Two different forms for a bending energy profile of a homopolymer. Shown is the (effective) bending energy per bending site *E*(*θ*). If the profile is purely convex down (black curve), the minimal energy conformations of the polymer is uniform bending (all sites are identically bent). If the function has a non-convex region (blue curve), non-uniform bending is more energetically favorable. In this case the total energy of the system follows the convex hull of the energy curve (red line).

Response of DNA to mechanical stress has been studied extensively [2, 8, 37, 40, 42–60], leading to a consensus in modeling the weak bending regime. Arguably the most widely used simplified model of DNA bending is the wormlike chain (WLC) model. In the original WLC [42, 61], the polymer is modeled as a continuous, isotropic elastic rod with its deformation energy being a quadratic function of the deformation angle. In the discrete version of WLC model, the bending energy of the polymer consisting of *N* segments of length *l* is given by:

(1) $$E_{\text{chain}}=\overset{N-1}{\underset{i}{\sum }}\frac{1}{2}k_{B}T\frac{L_{p}}{l}\theta_{i}^{2}$$

where *θ*_*i*_ is the angle between two consecutive segments (see inset of Figure 1). While this simplistic model lacks some features of the real DNA, such as sequence dependence of its local mechanical properties, it nevertheless captures the key physics of weak polymer bending, which explains why the model is robust and is widely adopted to interpret experiment. Various theoretical models of DNA bending, including those that explicitly account for the sequence-dependence [62–65], were consequently developed that also assumed harmonic (quadratic) angular deformation energy of DNA. There is very little doubt that the Hookean, “elastic rod” models accurately describe many polymers in the weak bending regime [1], including the double-stranded DNA [42, 54]. Indeed, lowest order term of a Taylor series expansion of any well-behaved function around its local minimum is quadratic, which means that for small deviations from equilibrium, the response function can be considered harmonic. However, by the same logic it should be expected that beyond a certain threshold the bending energy may no longer be approximated by the quadratic term alone; investigations of possible influence of non-harmonic terms on the mechanical properties of double-stranded DNA is a relatively new area. Historically, only very large fragments (hundreds to thousands of base-pairs) were investigated [37, 38], which are well-described by the WLC regardless of what happens on short length-scales [48].

However, within the last decade or so, the prevailing view of DNA as a Hookean polymer was challenged by experiments that were able to investigate the flexibility of DNA on scales smaller than several *L*_*p*_. Counter-intuitively, small DNA fragments (≈ 100 bp) were found to have much higher probability of cyclization (spontaneous formation of loops) than that predicted by the WLC theory [52]. This discovery sparked a debate, including subtle issues related to the interpretation of critical cyclization experiments themselves [35]. The controversy surrounding the DNA softening at short length scales remains unresolved, despite much effort. What is particularly puzzling is that strongly bent DNA appears *less* rigid than the DNA in the Hookean regime. Some of the follow-up experimental and theoretical work supports the validity of WLC even for tightly bent DNA [20, 42, 66], while others still show that short, strongly bent DNA is much more flexible [19, 48, 67, 68] than previously thought, in a manner that can not be described by a harmonic model [48]. A number of theoretical models have been proposed to account for the unexpectedly high flexibility of strongly bent double-stranded DNA. One popular model [69]—the meltable WLC or MWLC—postulated that the extra flexibility stems from formation of small local “bubbles” of single stranded DNA, which is much softer than the double helix. However, the degree of softening provided by the mechanism was later found [70] to be inadequate to fully explain the very sharp bends in DNA observed experimentally; in atomistic simulations, negative super-coiling was required to induce such bubbles in DNA mini-circles [71]. An early model [72], put forward well before the unusual DNA flexibility was discovered experimentally, suggested that the energy of a bent double-helix could be lowered by formation of sharp, ~ 90° kinks that maintain the Watson-Crick pairing along the helix. Sharp kinks were indeed observed in a pioneering atomistic simulation [53] some 30 years later, but subsequent improvements in the simulation methodology indicated that these were only induced at a high bend angle equivalent to those occurring in circles of just 45 base-pairs [73], while experimental softening of the DNA is seen experimentally for circles as large as ~ 106 base-pairs [19]. Sharp kinks in double-stranded DNA can be introduced empirically into the WLC model, e.g., by adding freely-bending hinge elements to the WLC chain, leading to a kinkable WLC, or KWLC [49]. A non-linear empirical bending potential that allows for the possibility of ~ 90° kinks in double-stranded DNA was recently proposed [20], but its physical origins, the critical value of the DNA curvature at which the kink occurs, and the corresponding energy gain remained unknown [20]. At the same time, a purely linear empirical bending potential was shown [48] to describe the softer DNA seen in AFM experiments, although the origin of the linear regime and its parameters (e.g., critical bend angle where the linear regime begins) remained unclear. Are the kinking and the linear regime just two manifestations of a deeper underlying principle?

In summary, the nature of the effective bending energy of double-stranded DNA in the strong bending regime, and importantly, the precise connection to the observed softening of the polymer is not fully clear. The influence of mechanical constraints on this connection remains unexplored. It is unclear which aspects of the DNA structure and interactions at atomic level are sufficient to explain the softening of strongly bent DNA. The authors find it hard to believe that very special models are needed to describe strong bending of the DNA; rather it is more likely that the curious case of the DNA is just a special case of a broader underlying theory applicable to all polymers. This work is an attempt to develop the backbone of one such a theory.

In this work we propose, and verify against available experiment, a unified theoretical description of polymer bending that treats the weak and strong bending regimes on the same footing, guided by a simple physical principle. The proposed framework does not rely on *ad-hoc* postulates; instead, it shows how the apparent softening of strongly bent DNA follows naturally from a specific mathematical property of the bending energy. Simulations suggest an atomistic explanation for the specific shape of the bending energy function.

## 2. METHODS

### 2.1. DNA Bending Energy From Experimental Data

A statistically significant, diverse set of several hundred PDB structures of protein-DNA complexes was investigated previously in reference [42]. The probability distribution of the experimental DNA bending angles (more than 10,000 values) was used in reference [42] to approximate the bending energy *E*(*θ*) [per base pair] as a fourth order polynomial: *E*(*θ*) = 203.1*θ*^2^ − 552.7*θ*^3^ + 416.8*θ*^4^ (where *θ* is in radians and *E*(*θ*) is in units of *kT*). Here we use this *E*(*θ*) to represent the experimental effective bending energy of the double-stranded DNA, blue line in Figure 4.

### 2.2. Atomistic MD Simulations of Closed DNA Loops

To avoid end effects, and make a close connection with DNA cyclization experiments, we employed closed DNA circles to estimate their effective bending energy *E*(*θ*) per base pair. DNA circles of various sizes, from 50 to 400 bp, were generated using NAB [74] (AmberTools) for sequence poly(dA).poly(dT), helical repeat of *h* = 10 bp, and other parameters of B-DNA as specified in NAB. We deliberately chose this simple, uniform sequence to focus on the basic physics of DNA deformation. The range of circle sizes was determined by (1) the ability to simulate meaningful time-scales (large circles), and (2) the limitations of NAB software to construct highly bent structures (small circles). To minimize torsional stress, only circles with an integer number of helical repeats were constructed.

All of the atomistic MD simulations were performed within AMBER-12 package, using ff99bsc0 force-field. The Generalized Born (GB-HCT, AMBER option igb=1) implicit solvation model was used to treat solvation effects, including the effects of 0.145M of monovalent salt. No long-range cut-off was employed. The model’s performance in atomistic simulations of DNA, including studies of its deformation [51], is well-established [75]. Two critical advantages of the implicit solvation approach over the more traditional explicit solvation [76] treatment made the implicit solvation the method of choice in this work. These advantages are the superior simulation efficiency for large DNA structures [77] and the straightforward manner in which their energies, including free energy of solvent re-arrangement, can be estimated [75] within the implicit solvation framework.

All DNA circles were initially minimized for 1,000 steps with “P” atoms constrained to the original positions with a force constant of 1.0 kcal/mol/Å^2^ to enforce the circular shape. Each system was then heated to 300 K and equilibrated for 100 ps with the same restraints as for the minimization. Shake was used to constrain the hydrogen atoms; we employed 2 fs time-step for the atomistic simulations. Finally, we generated 1 ns long MD trajectory for each circle at 300K, with “P” atoms also restrained with a force constant of 0.1 kcal/mol/Å^2^, sufficient to support the near perfect circular shape of the fragment, but allowing for local re-arrangements. The energies and their components, including the electrostatic, VDW, bond, etc. were saved every 20 fs, and averaged over the whole trajectory. The relatively short simulation time allowed us to simulate even the largest of the circles; it is justified by the use of the strong positional restraints, which permit only local, very fast structural re-arrangements. For smaller circles we verified that increasing the simulation time by an order of magnitude had negligible effect on the computed averages. In Figures 6, 7, the energy per bp was computed as the difference between per bp potential energies of the given circle and the largest circle simulated, in which the DNA was virtually unbent.

### 2.3. Coarse-Grained Simulations of DNA Loops

**FIGURE 2 |:**
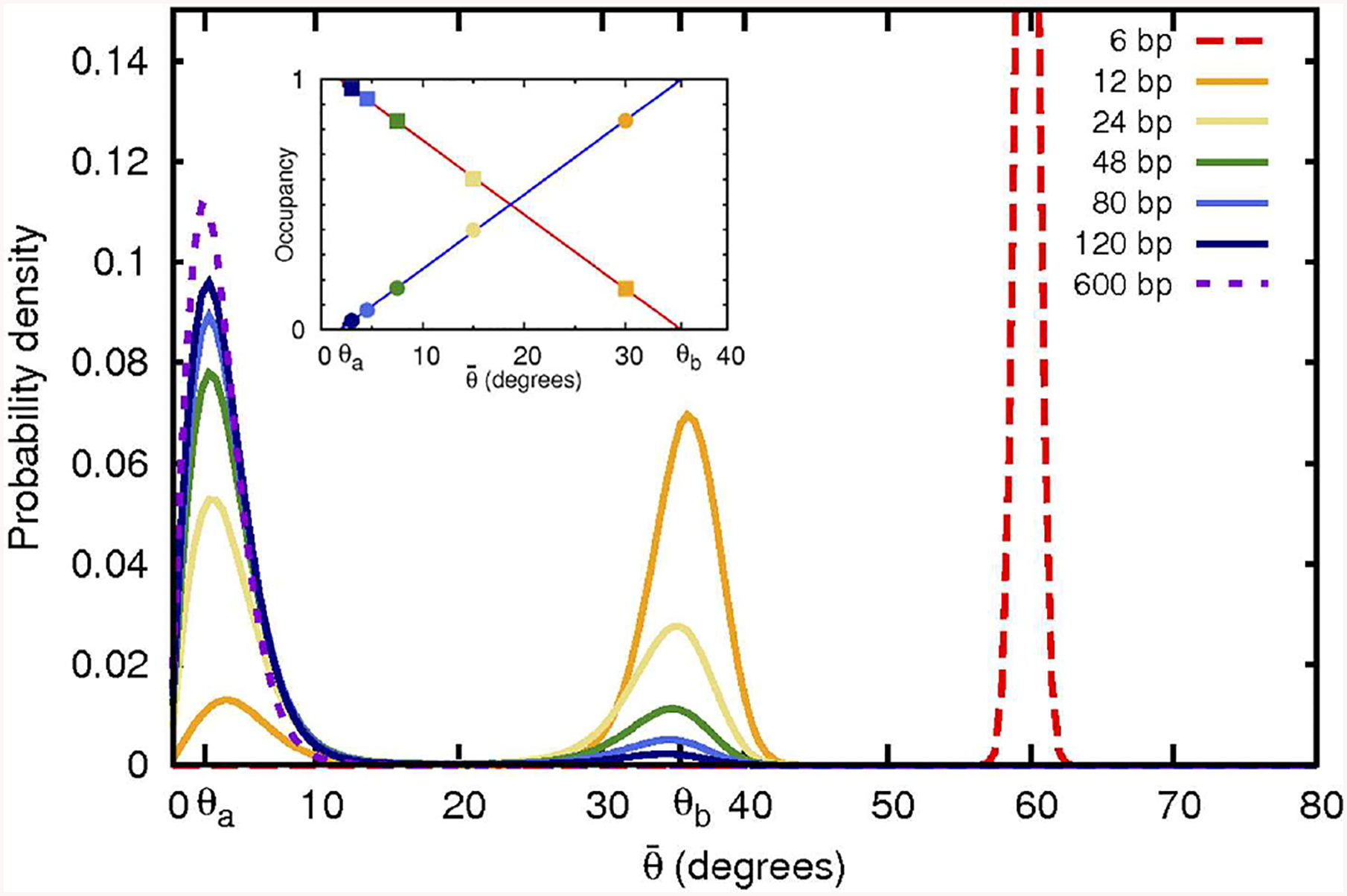
Non-convex bending energy function leads to bi-modal distribution of bending angles. Shown are angular probability distributions at 300 K resulting from a realistic non-convex bending potential (Figure 4) used here in coarse-grained simulations of DNA closed loops of variable size. As the loop size (indicated in the top right corner) decreases, the average bending angle per base pair increases. When the average angle falls into the convex hull range, the angular distribution becomes bi-modal with peaks at *θ*_*a*_ and *θ*_*b*_, corresponding to the weakly and strongly bent states, respectively. Fractional occupancy of both of these states of bending is shown in the inset as a function of the average bend angle $\bar{\theta }$. Squares: occupancy of the weakly bent state. Circles: occupancy of the strongly bent state, which can be interpreted as a “kink.” Out-of-plane motion likely affects angular probability distribution of the largest (600 bp) loop, which may explain the shift, compared to expectation, of the position of the corresponding distribution peak.

**FIGURE 3 |:**
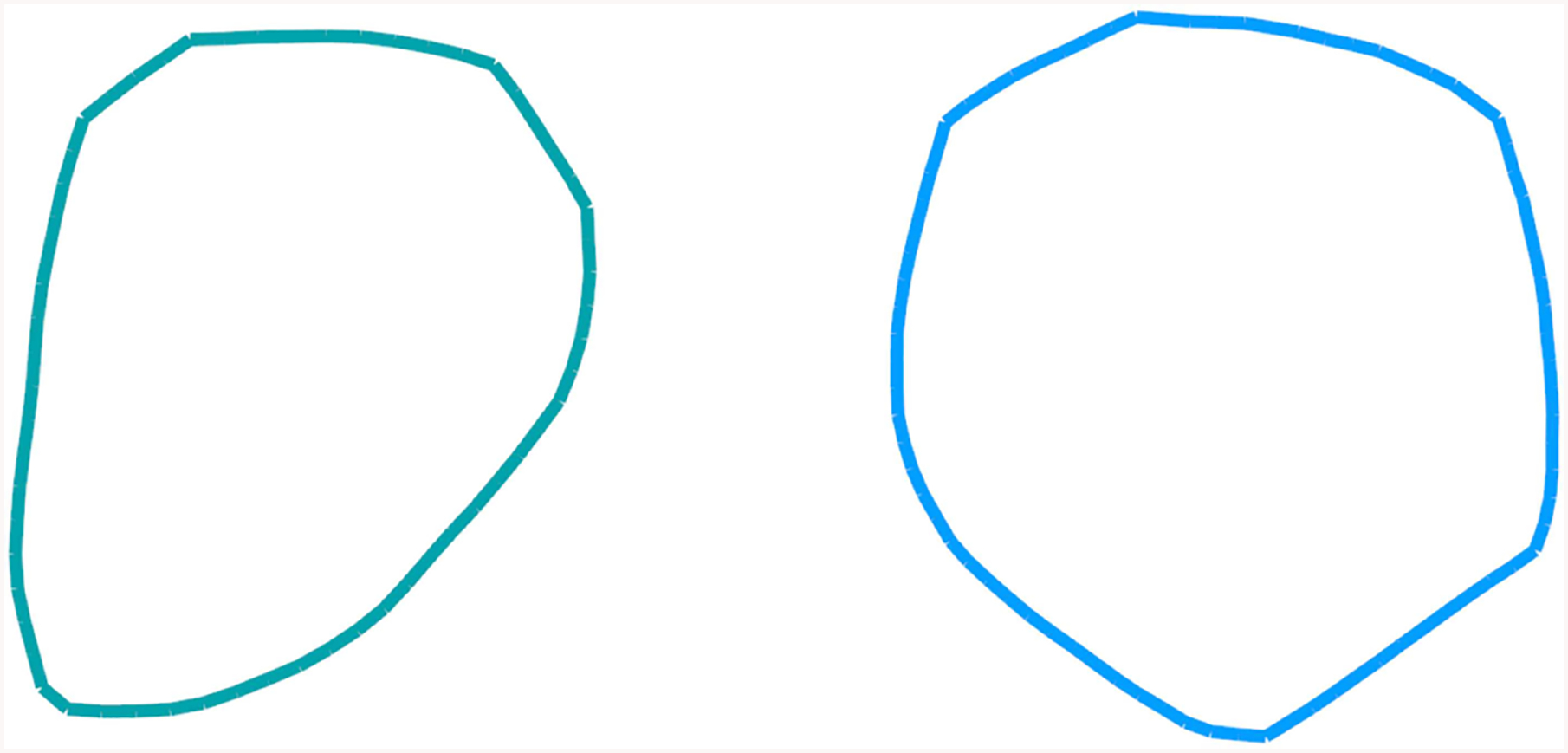
Examples of a 60- base-pair (left) and 80- base-pair (right) loop conformations at 300 K corresponding to the non-convex bending potential of Figure 4. The conformational ensembles were generated via coarse-grained simulations of DNA closed loops.

**FIGURE 4 |:**
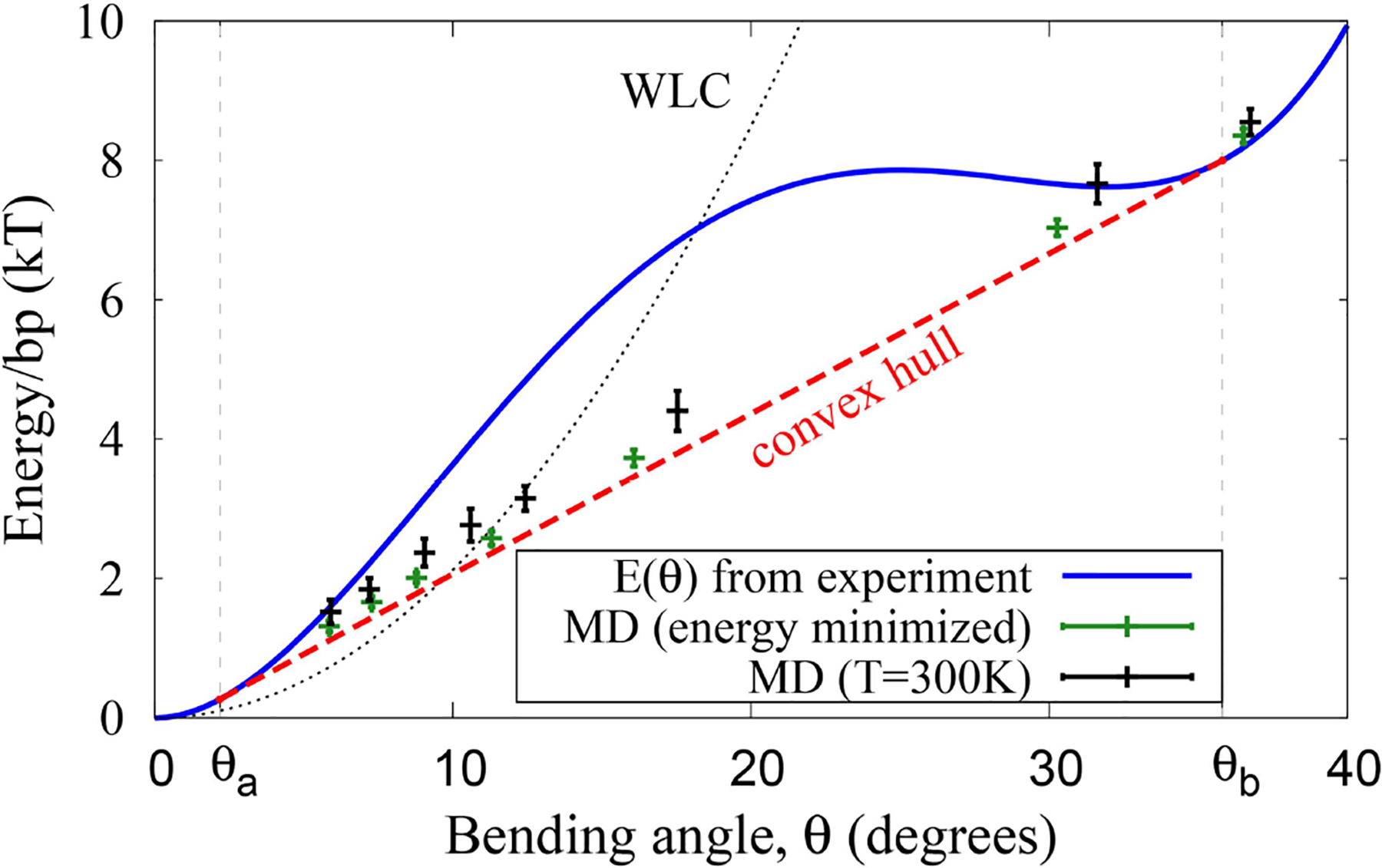
DNA effective bending energy *E*(*θ*) (per bp) extracted from the probability distribution [42] of DNA bends that naturally occur in protein-DNA complexes (blue line), and the average energy of unrestrained DNA closed loops simulated via coarse-grained MD with the same *E*(*θ*) (crosses). Green symbols: energy minimized (simulated annealing) loops. Black symbols: loops simulated at T=300K. In both cases, the average loop energy as a function of average bend angle $\bar{\theta }=\theta$ follows the convex hull of *E*(*θ*). The small deviation of the T = 300 K points from the convex hull are a result of ensemble average sampling and insignificant out-of-plane bending seen in the simulation.

ESPResSo [78] was used to create and simulate coarse-grained closed loops of DNA of different sizes, from 6 to 600 bp long. A single bead of the appropriate mass represents one base-pair of B-DNA; the bead-bead distance was set to 3.3 Å, corresponding to the average distance between base pairs in canonical DNA. The bonds between the beads were made virtually inextensible (very large coefficient of the quadratic bond stretching energy); the bond angle potential (effective bending energy) between neighboring beads was defined to have the same form as in Figure 4, that is correspond to the bending potential inferred from the experimental data [42]. No further bead-bead interactions or constraints on the loop geometry were imposed. The loops were simulated at *T* = 300*K* to generate 100,000 snapshots for each loop size. The resulting bend angle distributions are shown in Figures 2, 3. Steepest descent minimization of the last snapshot from each distribution was used to obtain the “energy minimized” data points shown in Figure 4.

### 2.4. Coarse-Grained Simulations of Confined DNA Fragments

#### 2.4.1. Protein-DNA Complex

ESPResSo [78] was used to create and simulate a 20 bead long fragment of “DNA” bound to a spherical charged “protein” (Figure 8). The beads and their interactions were set up as described above, with the following modifications. The end beads were not linked to create a loop. Each bead carried a unit charge *q*_*s*_ = −1 (atomic units); The bead charges interacted only with a positive charge *Q* of the “protein,” represented by a spherical impenetrable constraint of radius *R*. In addition, two impenetrable walls were placed above and below the charge *Q* to minimize out-of-plane bending of the “DNA.” The confining charge *Q* was varied from 10 to 1,000, effectively sampling two orders of magnitude of confinement strength (defined here as |*Q*/*q*_*s*_|). The constraint radius *R* was also varied to sample various curvature values of the “protein,” and thus various total bending angles of the confined “DNA,” Figure 8.

#### 2.4.2. A Nucleosome Model

For the nucleosome model, the system described above was modified to mimic the confinement of DNA around realistic histone core. The DNA fragment size was increased to 147 bp, and non-bonded interactions between monomers were turned on for an additional realism [79]. The fragment was confined around a cylinder of fixed diameter *R* ≃ 100Å, and the walls were placed ~50Å apart (approximate dimensions of the nucleosome complex [79]).

## 3. RESULTS AND DISCUSSION

### 3.1. The Proposed Unified Framework of Polymer Bending

We begin with a useful analogy from classical thermodynamics that connects system’s stability to convex properties of its governing potential. For example, for a system to be stable against a macroscopic fluctuation in energy, the entropy of the system as a function of energy, *S*(*E*), must be concave (non-convex). Any chord connecting two points on a graph of *S*(*E*) must lie below the curve itself in order to satisfy the second law of thermodynamics (maximum *S*). Conversely, the inverse function *E*(*S*) must be convex. If, however, *E*(*S*) is not convex over some region, the system phase-separates once this region is reached, with the properties of the two phases corresponding to the end points of the convex hull of the non-convex region. The actual, physical average energy of the system follows the convex hull, which makes the energy manifestly convex. This very general reasoning, with appropriate choice of the perturbation coordinate and potential, is applicable to phase transition of single species polymers (Flory-Huggins Theory [80]), as well as to stretching of polymers [21] and other materials [81]. Here we use the analogy to develop a general framework that describes polymer response to bending, weak and strong, on the same footing.

Consider a polymer chain made of *N* ≫ 1 inextensible, *identical* monomer segments with effective bending deformation energy *E*(*θ*_*i*_) for each bending site, where *θ*_*i*_ is the angle between two successive segments (see inset of Figure 1). Here we assume that the effective *E*(*θ*) takes into account all the interactions, short- and long- range, between the monomers. For notational simplicity, in what follows we ignore the difference between *N* and *N* − 1 for large *N*. The total energy of the polymer is $E_{\text{chain}}=\sum_{i}^{N}E\left(\theta_{i}\right)$, and without loss of generality we assume no intrinsic bends, i.e., *E*(0) = 0. Just like in WLC, we assume isotropic bending energy, which is a reasonable assumption for DNA fragments longer than 2 helical repeats or 20 bp [20]. For the moment, we further assume no torsional degrees of freedom. In order to induce an average non-zero bend in the chain, the polymer must be constrained, and the problem of finding the equilibrium polymer conformation is reduced to minimizing *E*_*chain*_, subject to the specific constraint of the problem. Here we assume that entropic effects are relatively small at length scales of interest (≲ *L*_*p*_)—an assumption that we explicitly confirm below by numerical experiments. We begin by considering a very special case of a uniformly bent polymer—constrained to have the same constant curvature along the entire chain. By construction, such a polymer consists of identically bent segments with each bending angle *θ*_*i*_ equal to the average deformation angle, $\bar{\theta }=N^{-1}\sum_{i}^{N}\theta_{i}$, and its total energy is $NE(\bar{\theta })$. Uniformly bent DNA circles at atomic resolution will be used further in this work to analyze physical origins of the specific shape of $E(\bar{\theta })$.

Next, consider a more realistic situation where the polymer bending is enforced by a much less restrictive constraint: that the sum of the bend angles between the monomers remains constant, $\alpha =\sum_{i}^{N}\theta_{i}=\text{const}$. Note that this constraint alone does not fully define the geometry of the polymer. A closed planar loop, with the first and last segments linked, is a relevant example for which the constraint is satisfied; $\sum_{i}^{N}\theta_{i}=2\pi$, from elementary geometry of polygons (see also the Supplementary Material). Mathematically, the problem of finding the minimum energy conformation of the polymer is that of energy minimization under the specific constraint:

(2) $$E_{\text{chain}}=NE(\bar{\theta })=\underset{\sum_{i=1}^{N}\theta_{i}=N\bar{\theta }=\alpha }{\text{min}}\left\{\overset{N}{\underset{i}{\sum }}E\left(\theta_{i}\right)\right\}$$

where we make a clear distinction between E, which is the average bending energy per bending site *in the minimum energy state* of the polymer, and *E* corresponding to the uniform bending. Using Lagrange multipliers, Equation (2) can be reduced to min{*E*(*θ*_1_)+ ⋯ +*E*(*θ*_*N*_)−λ(*θ*_1_+ ⋯ +*θ*_*N*_−α)}. Differentiating with respect to *θ*_*i*_ gives a set of equations $\partial_{\theta_{i}}E\left(\theta_{i}\right)-\lambda =0$ (for all *i*) which leads to a set of equalities $\partial_{\theta_{1}}E\left(\theta_{1}\right)=\partial_{\theta_{2}}E\left(\theta_{2}\right)=\cdots =\partial_{\theta_{N}}E\left(\theta_{N}\right)$. For a convex functional form of *E*(*θ*), ∂_*θ*_*E*(*θ*) monotonically increases with *θ*, and therefore the equalities are satisfied only if *θ*_1_ = *θ*_2_ = ⋯ = *θ*_*N*_: the polymer is always uniformly bent, that is each segment is bent through the same angle $\theta =\bar{\theta }$ and $E(\bar{\theta })=E(\bar{\theta })$ However, for a non-convex function, such as one shown in Figure 1, there can be more than one value of *θ* that satisfies $\partial_{\theta_{1}}E\left(\theta_{1}\right)=\partial_{\theta_{2}}E\left(\theta_{2}\right)=\cdots =\partial_{\theta_{N}}E\left(\theta_{N}\right):\partial_{\theta_{i}}E\left(\theta_{a}\right)=\partial_{\theta_{i}}E\left(\theta_{b}\right)$ for some *θ*_*a*_ < *θ*_*b*_.

Of special importance are *θ*_*a*_ and *θ*_*b*_ that mark the beginning and the end of the convex hull of *E*(*θ*)—the segment of a straight line tangent to the non-convex function at two points, such that for any argument between these two points the value of the function at the argument is greater than that of the convex hull line at the same argument (Figure 1). For bend angles $\bar{\theta }$ in the convex hull interval, $\theta_{a}<\bar{\theta }<\theta_{b}$, a uniformly bent chain is no longer the stable minimum energy conformation of the polymer. Instead, the stable minimum is achieved when the distribution of bend angles is *bi-modal*: each segment is bent through one of the two bending angles *θ*_*a*_ or *θ*_*b*_. To see how this “phase separation” comes about, consider a uniformly bent conformation of a polymer with just two sites, and let us investigate its stability to perturbation. A perturbation Δ*θ* that reduces the bend angle at one site means that the other bending site must increase its bend angle by Δ*θ* in order for the total bend to remain unchanged, that is to satisfy the constraint $N\bar{\theta }=\text{const}$. This perturbation changes the total energy *E*_*chain*_ by $E(\bar{\theta }+\Delta \theta )+E(\bar{\theta }-\Delta \theta )-2E(\bar{\theta })$. If *E*(*θ*) is a convex function (e.g., the black curve in Figure 1), the perturbed system will have a higher energy than in the initial uniformly bent case. This is because, by definition, a convex curve always lies below its chords, so that $2E(\bar{\theta })<E(\bar{\theta }+\Delta \theta )+E(\bar{\theta }-\Delta \theta )$. Thus, the perturbation leads to an increase in system’s energy, which implies that the system was at a stable equilibrium. Therefore, the minimum energy conformation of a polymer with a convex effective bending energy is always that of a uniformly bent chain. However, if the function *E*(*θ*) has a non-convex region (e.g., the blue curve in Figure 1), then for any $\bar{\theta }$ in that region, and Δ*θ* that does not take the system outside of it, $2E(\bar{\theta })>E(\bar{\theta }+\Delta \theta )+E(\bar{\theta }-\Delta \theta )$, which means it is possible to lower the energy of the polymer further by non-uniform bending. Namely, one site is now bent through $\bar{\theta }-\Delta \theta$, and the other through $\bar{\theta }+\Delta \theta$, with the new value of the average chain energy per site, $\frac{1}{2}E_{\text{chain}}$ falling on the midpoint of the line (dashed red line in Figure 1) connecting the two new bending states on the energy curve. The longer the chord connecting the two perturbed states at $\bar{\theta }-\Delta \theta$ and $\bar{\theta }+\Delta \theta$, the larger the energy gain $2E(\bar{\theta })-E(\bar{\theta }+\Delta \theta )-E(\bar{\theta }-\Delta \theta )$ due to the non-uniform bending, for as long as the chord is completely below the *E*(*θ*) curve. The largest energy gain, and thus lowest possible *E*_*chain*_, is achieved for the limiting chord that is the convex hull of *E*(*θ*)—the line segment tangent to the non-convex function at two points, such that between these two points the value of the function is greater than that at any point of the line segment. For this limiting case, $\bar{\theta }-\Delta \theta =\theta_{a},\bar{\theta }+\Delta \theta =\theta_{b}$.

Thus, one clear and testable consequence of non-convexity of the bending potential (Figure 1), is that the corresponding distribution of polymer bend angles becomes bi-modal once the average bending angle $\bar{\theta }$ is within the convex hull region. A weak enough bending is always uniform, for as long as the average bend angle $\bar{\theta }$ is below *θ*_*a*_. As the average bend angle becomes just slightly larger than *θ*_*a*_, most of the segments are still bent weakly through *θ*_*a*_, but a small fraction becomes strongly bent through *θ*_*b*_. As the constraint forces the system to bend further, the fraction of the strongly bent segments increases linearly with $\bar{\theta }$, until, eventually all the segments are strongly bent through *θ*_*b*_. Beyond that point the system re-enters the uniform bending regime again. Here we confirm this expectation quantitatively (Figure 2), within a coarse-grained DNA model with one bead per bp (see “Methods”). Specifically, we have analyzed angular distribution of different sized loops, from 6 to 600 bp long. The corresponding probability distributions of bend angles for each loop size are shown in Figure 2, with examples of structures from these distributions presented in Figure 3. Note that for a large, but finite *N*≫1, the predictions in Figure 2 should be interpreted as applicable to the entire conformational ensemble of bent loops; in particular, the predicted linear dependence of the fractions of strongly and weakly bent fragments apply to the ensemble averages. For a given value of $\bar{\theta }$, between *θ*_*a*_ and *θ*_*b*_, one observes a distribution of tightly bent fragments among the loops, a given loop can have none or more than one; bent conformations we have observed in our simulations are qualitatively consistent with the above picture. In this work we are not pursuing a detailed analysis of many “structural” consequences of the proposed model; we hope to revisit this issue in the future. In what follows we focus on the energy aspects of the model, which can be tested against DNA cyclization experiments.

We begin by deriving an explicit expression for E(θ) for $\theta_{a}<\bar{\theta }<\theta_{b}$. In the minimum energy conformation, let 0 < *p* < 1 represent the fraction of all the bending sites that are in the state *θ*_*b*_ and 1−*p* the fraction of the remaining sites in the state *θ*_*a*_. The total bending angle in terms of *θ*_*a*_ and *θ*_*b*_ is then given by $Np\theta_{b}+N(1-p)\theta_{a}=N\bar{\theta }=\alpha$, and the bending energy per monomer in the non-convex region is $E(\bar{\theta })=pE\left(\theta_{b}\right)+(1-p)E\left(\theta_{a}\right)$. Rewriting $p=\left(\bar{\theta }-\theta_{a}\right)/\left(\theta_{b}-\theta_{a}\right)$, we arrive at

(3) $$E(\bar{\theta })=\frac{\bar{\theta }-\theta_{a}}{\theta_{b}-\theta_{a}}\left(E\left(\theta_{b}\right)-E\left(\theta_{a}\right)\right)+E\left(\theta_{a}\right)$$

Therefore, in the non-convex region, the actual polymer energy per bending site, $E(\bar{\theta })$ corresponding to the stable minimum energy state, is a linear function of the *average* deformation $\bar{\theta }$. Clearly, $E(\bar{\theta })<E(\bar{\theta })$ within the convex hull interval (Figure 1).

To arrive at a general theory that can account for both the weak and strong bending regimes simultaneously, we use the form of Equation (3) for the strong bending regime, while retaining WLC for the weak bending. In the proposed Energy Convex Hull(ECH) model, the average per segment (e.g., per base-pair) bending energy is described by an everywhere differentiable piece-wise polynomial function: quadratic WLC (Equation 1) for $\bar{\theta }<\theta_{a}$, and a linear function—convex hull of *E*(*θ*)—for $\theta_{a}<\bar{\theta }<\theta_{b}$:

(4) $$E(\bar{\theta })=\{\begin{array}{ll} \frac{1}{2}k_{B}TL_{p}{\bar{\theta }}^{2} & \text{ if }\bar{\theta }\leq \theta_{a}\\ k_{B}TL_{p}\theta_{a}\left(\bar{\theta }-\frac{1}{2}\theta_{a}\right) & \text{ if }\theta_{a}<\bar{\theta }<\theta_{b} \end{array}$$

where *L*_*p*_ is the accepted persistence length, well-established for the weak bending regime; here it is dimensionless, expressed in terms of the number of bending sites (e.g., number of base pairs for DNA loops). In this work we are not interested in the extreme strong bending regime $\bar{\theta }>\theta_{b}$, since for the DNA this regime would correspond to loops smaller than 10 bp. Such small loops are likely physically impossible due to steric constraints, and are much smaller than those observed in cyclization studies [19, 82]. Thus, the only key parameter that ECH theory inherits from the input effective bending energy, *E*(*θ*) in Figure 4, is the value of *θ*_*a*_, which enhances robustness of the theory to inevitable imperfections [42] of the input bending energy profile. For example, a uniform re-scaling *E*(*θ*) → λ*E*(*θ*) would leave the x-coordinates *θ*_*a*_ and *θ*_*b*_ of the convex hull double-tangent segment unchanged because the derivatives would be re-scaled by the same λ. Further discussion of the robustness of ECH model to its parameters can be found below and in Supplementary Material.

### 3.2. Bending of a Circular Loop, Weak, and Strong

While many different types of constraints can be physically realized, one of the most important ones is the closed loop constraint, which is also used in DNA cyclization experiments [40, 42, 52], critical [49] for investigating the strong bending regime. Consider the case of a single closed loop $\alpha =\sum_{i}^{N}\theta_{i}=2\pi$. From Equation (4), the total bending energy of a closed loop of total length *L* (here *L* = number of base pairs in the loop, corresponding to *N* in Equation 2) is given by $E_{\text{loop}}=E(\bar{\theta })L$. Since $\bar{\theta }=\frac{\alpha }{L}=\frac{2\pi }{L}$, the bending energy of the loop is:

(5) $$E_{\text{loop}}(L)=\{\begin{array}{ll} 2\pi^{2}k_{B}T\frac{L_{p}}{L} & \text{ if }L>\frac{2\pi }{\theta_{a}}\\ k_{B}TL_{p}\theta_{a}\left(2\pi -\frac{1}{2}L\theta_{a}\right) & \text{ if }\frac{2\pi }{\theta_{b}}<L<\frac{2\pi }{\theta_{a}} \end{array}$$

The crucial difference between WLC and ECH is for small loops. Within WLC, the energy cost of bending a straight rod into a circular ring grows without a bound as the loop size decreases, consistent with a macroscopic intuition that a long rubber rod is much easier to bend into a circle than a short one. In contrast, within ECH the loop bending energy approaches a constant for small loop sizes.

Note that, where defined, the new function $E_{\text{loop}}$ depends explicitly on just one new parameter: *θ*_*a*_—lower boundary of the non-convex domain. In other words, as long as *θ*_*b*_ is large enough, its value does not affect $E_{\text{loop}}$. Although we tacitly assumed the loop to be confined to a 2D plane to simplify the derivations, our unconstrained coarse-grained simulations of closed loops at 300 K demonstrate (Figure 4), that the assumption has little effect on our key conclusions.

### 3.3. Application to Double-Stranded DNA

The preceding discussion was not restricted to the case of DNA: non-uniform, two-state bending, and the corresponding linear bending regime can be a feature of any polymer. However, since DNA is an important example from various perspectives, and since it exhibits tight looping in many different biological systems, we will focus on double-stranded DNA for the rest of the study. An effective bending energy (per bp) calculated from a statistical analysis of experimental PDB structures of DNA-protein complexes [42] is shown in Figure 4. This effective bending energy function has a non-convex region, and thus a convex hull, the end points of which are *θ*_*a*_ = 2.2° and *θ*_*b*_ = 35.8° (0.038 and 0.62 rad, respectively in Equation 5), corresponding to fragment lengths of *L* ~ 160 and ~ 10 bp, respectively for DNA closed loops. As a numerical example, the bending energy of a 50 bp circular loop is ~ 54*k*_*B*_*T* within WLC vs. ~ 30*k*_*B*_*T* within ECH. Coarse-grain molecular dynamics simulations at 300 K demonstrate that a polymer with this effective bending energy between monomers exhibits all of the key features discussed above (Figures 2, 4). For large loop sizes the bending angles are small (weak bending)—the system samples the convex (harmonic) region of the energy function (Figure 4), and the distribution of bend angles is uni-modal. However, as the loop size decreases, the average angle per bending site $\bar{\theta }$ increases, eventually crossing the *θ*_*a*_ threshold. Once this happens, the energy of the system per bending site increases linearly with $\bar{\theta }$, and the distribution of bend angles becomes bi-modal (Figure 2), until the system reaches the upper boundary of the convex hull at *θ*_*b*_.

### 3.4. Comparison With DNA Cyclization Experiments

**FIGURE 5 |:**
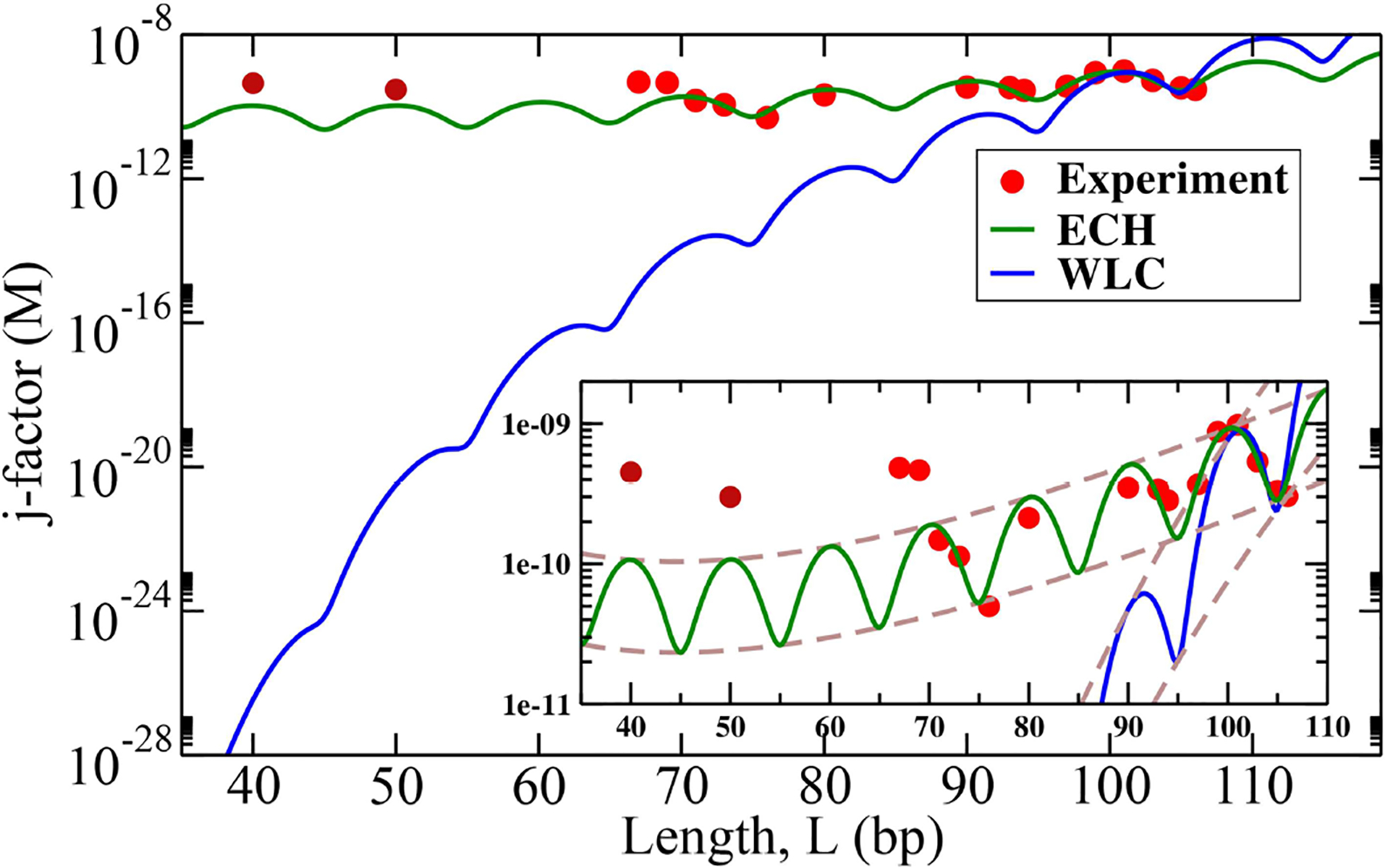
DNA cyclization *j-factors* computed using the proposed ECH model (green line) and WLC (blue line) are compared with recent experiment [19] (red dots, *L* > 60 bp). Experimental values of persistence length, *L*_*p*_ = 150 bp and *θ*_*a*_ = 2.2° (Figure 4) were used; the value of *k* in Equation (6) was obtained independently for each model as best fit against two experimental data points for fragment length L = 101 and 106 bp (see Supplementary Material). The envelopes of the *j-factor* (brown dashed lines) for ECH and WLC are shown in the inset. Predicted envelope for ECH *j-factor* has a minimum near 45 bp. The experimental data points *L* = 50 and *L* = 40 bp were shared by Taekjip Ha (see reference [19]) in private communication to assess model performance *after* the model had been constructed.

Most experimental cyclization results are expressed [42, 52] in terms of the Jacobson-Stockmayer *j-factor*, which estimates the probability that a linear polymer of length *L* forms a closed loop by joining its cohesive ends [40, 83]. Here we use the well-established [84, 85] Shimada-Yamakawa formula for the *j-factor* of a closed loop:

(6) $$j(L)≃\frac{k}{L_{p}^{3}}{\left(\frac{L_{p}}{L}\right)}^{5}\text{exp }\left(-\frac{E_{\text{loop}}}{k_{B}T}+\frac{L}{4L_{p}}\right)$$

where $\frac{1}{L_{p}^{3}}{\left(\frac{L_{p}}{L}\right)}^{5}\text{exp }\left(\frac{L}{4L_{p}}\right)$ accounts for the entropic contribution [85], from possible looping geometries, and $\text{exp }\left(-\frac{E_{\text{loop}}}{k_{B}T}\right)$ is the energy penalty of bending the DNA fragment to form the loop of optimal geometry. We note that *k* in the above expression depends, in a complex manner, on the loop closing geometry, but can be expected to remain invariant over a relatively short range of loop lengths *L*, within the same experiment. The same procedure (see the Supplementary Material), is used to obtain the best fit for *k* for both ECH and WLC, thus the details subsumed in *k* effectively “cancel out” in the comparison, which allows us to focus on qualitative, orders-of-magnitude, differences between the two theories. While Monte-Carlo based numerical approaches to computing *j-factor* exist [23, 25, 85], in this proof-of-concept work we prefer a closed-form analytical expression, which can be easily explored in various regimes. For loop sizes within our range of interest (Figure 5), the Shimada-Yamakawa formula was shown [85] to approximate the corresponding numerical estimate fairly closely. To make a direct connection with cyclization experiments for non-integer numbers of helical repeats, we modulate the torsionally independent loop energy from Equation (6) with *cos*(2*πL*/*h*), where we assumed the helical repeat *h* = 10 bp per turn. The agreement with the cyclization experiment is robust with respect to the precise value of the helical repeat (see Supplementary Material). This form of the modulating factor is adopted from reference [84] to account for the periodic variation of the *j-factor* due to the torsional component of the energy [84]. This simple way of accounting for non-integer numbers of helical repeats is appropriate [25] for the type of cyclization experiment [19] we use as reference [25]; the approach is sufficient for the purpose of testing key predictions of ECH vs. WLC, its simplified nature does not affect the comparison with the overall (envelope, average) behavior [49] of experimental *j-factors* (see also Table 1 below). We use $E_{\text{loop}}(L)$ defined in Equation (5) for ECH and $E_{\text{loop}}(L)=2\pi^{2}k_{B}T\frac{L_{p}}{L}$ for all *L* in the case of WLC. The proposed ECH model and WLC are compared with the most recent experiment [19] in Figure 5.

As seen from Figure 5, ECH leads to a noteworthy agreement with the cyclization experiment, while the *j*-factors predicted by conventional WLC are off by several orders of magnitude in the strong bending regime (WLC is known to work well in the weak bending regime where it coincides with ECH by construction). The agreement of ECH with the experiment is robust to the value of its key input parameter *θ*_*a*_, see below and SI. The ratio of *j-factors* for various loop lengths—independent of *k* in Equation (6)—is predicted to be within 50% of the experiment, while the corresponding WLC predictions are up to six orders of magnitude off (Table 1). Note that ratios of *j-factors* at integer values of helical repeats can be predicted directly from Equation (6), which does not contain the oscillatory components.

#### 3.4.1. Cyclization of Very Short Loops

Counter-intuitively, the predicted envelope function for ECH *j-factor*, which is essentially Equation (6), has a minimum. While the minimum is rather broad (see the inset of Figure 5), it is well-defined and occurs at

(7) $$L=\frac{5}{\frac{1}{4L_{p}}+\frac{L_{p}\theta_{a}^{2}}{2}}$$

Using the value of *L*_*p*_ = 150 bp and *θ*_*a*_ = 2.2°, the minimum of *j-factor* is found at L ≃ 45 bp. No such minimum exists in the WLC case in the range of *L* of interest to us.

For loops even smaller than *L* ≃ 45 bp, the *j-factor* begins to increase, whereas for WLC *j-factor* decreases sharply for small loops. This completely counter-intuitive behavior of the cyclization probability for very tight loops predicted by ECH is borne out by experiment (Figure 5); its physical origin is explained below. The two experimental points at L = 50 and L = 40, which *qualitatively* support the counter-intuitive prediction of the theory, were not available to us until after the ECH framework was fully developed and tested against published data [19] for larger circles. The over-all variation of the *j-factor* as a function of the loop length for both models is governed by an interplay between the entropic and the mechanical bending energy costs $E_{\text{loop}}(L)$ of forming the loop. For small loops, the entropic penalty of forming the loop decreases with the loop size *L*; however, $E_{\text{loop}}(L)\sim L_{p}/L\to \infty$ for *L* → 0 within WLC, which leads to the steep decrease in the over-all cyclization probability (Figure 5). Note that the ~ *L*^−1^ divergence of WLC bending energy for small *L* is independent of the loop closure geometry [86, 87], including the “teardrop” not explicitly considered here. In contrast, ECH loop energy, Equation (5), approaches a constant for small *L*, which explains why the corresponding *j-factor* reaches a minimum and then begins to increase for small enough *L* (Figure 5). This very different qualitative behavior of WLC and ECH *j-factors* for small loops can be used as a discriminating experimental test of these two models. The existence of the minimum of *j-factor* for short loop sizes can be used to discriminate between models (e.g., references [25, 35, 48, 49, 69]), that account for the DNA softening at short length scales; the predicted minimum value of the *j-factor* can be used to further discriminate between those models that exhibit the minimum. For example, both KWLC [49] and a recent version [23] of MWLC predict a *j-factor* minimum, but the loop sizes at which the minima occur appear to be substantially different from the ~ 45 base pairs predicted by ECH. At the same time, a coarse-grained model [35] designed around basic structural, mechanical, and thermodynamic properties of single-and double-stranded DNA reveals a minimum in the *j-factor* at around ~ 45 base-pairs. While the absolute value of the predicted *j-factor* depends on exactly how the experiment is interpreted [35], the position of the minimum appears robust to these details.

Within the proposed ECH framework of polymer bending, the central role is played by convexity properties of the effective bending energy between individual monomers. For the DNA, we used the energy profile inferred from statistical analysis of experimental structures of protein-DNA complexes (Figure 4)—the energy has a clear non-convex region, responsible for the “softer,” linear bending mode of short DNA loops. The same general considerations will hold for any effective bending energy that has a distinct non-convex region regardless of its origin [25], including a kinkable WLC potential [20]. Thus, even though ECH explains experimental results perceived to be in contradiction with WLC, there is no fundamental contradiction between the new framework and the conceptual basis of WLC.

### 3.5. Origin of the Non-convex Bending Energy of DNA

**FIGURE 6 |:**
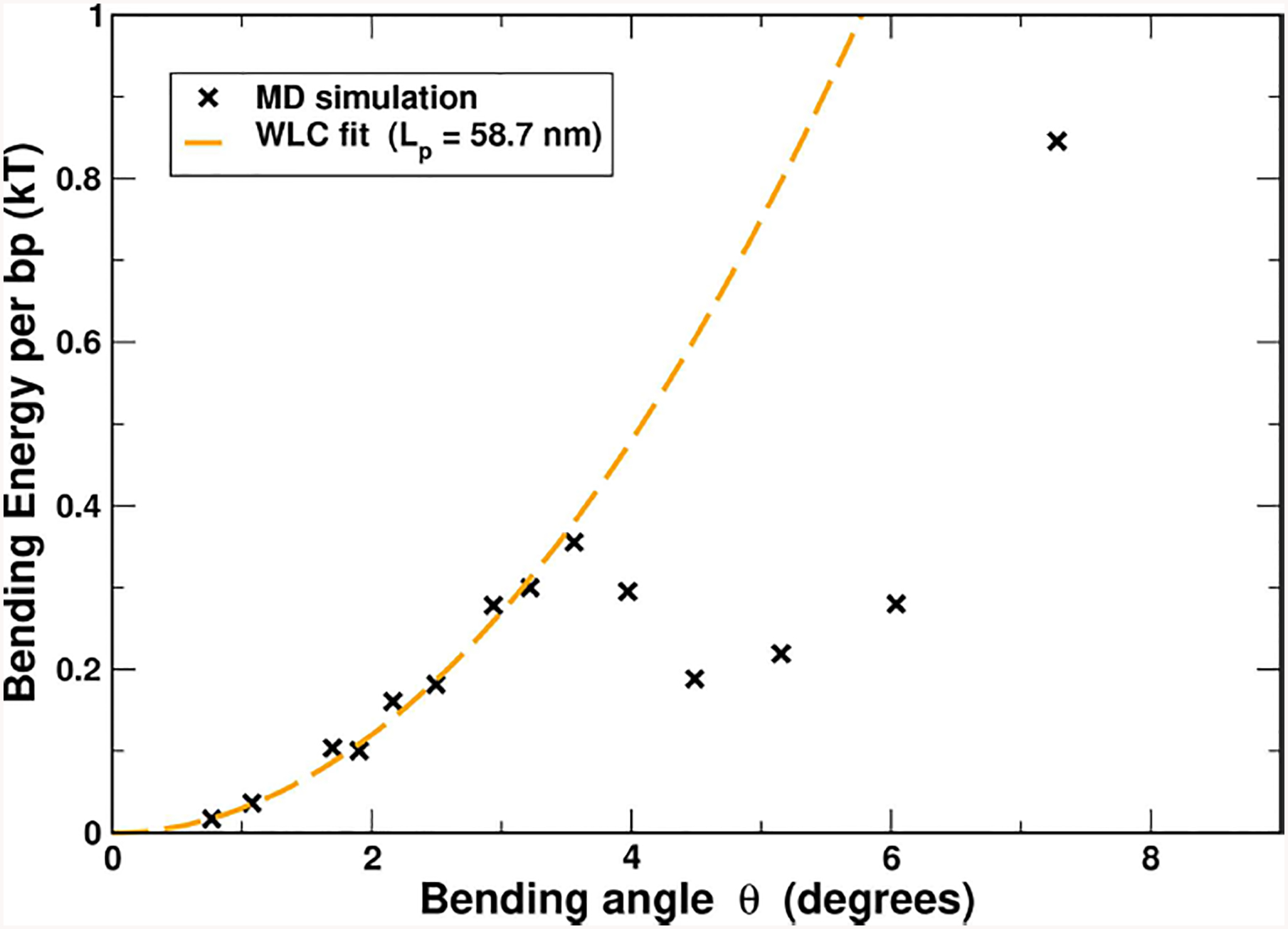
The effective DNA bending energy, per base pair, as a function of the bending angle *θ*, inferred from all-atom MD simulations of uniformly bent DNA circles of variable lengths (50–400 bp). The statistical error bar is smaller than the symbol size. For reference, a WLC fit for small angle bends (up to≈ 3.5°, dashed line) is shown; the fit yields a persistence length of 58.2 nm (≈ 172 bp), reasonably close to the experimental value of ≈ 50 nm (≈ 150 bp).

**FIGURE 7 |:**
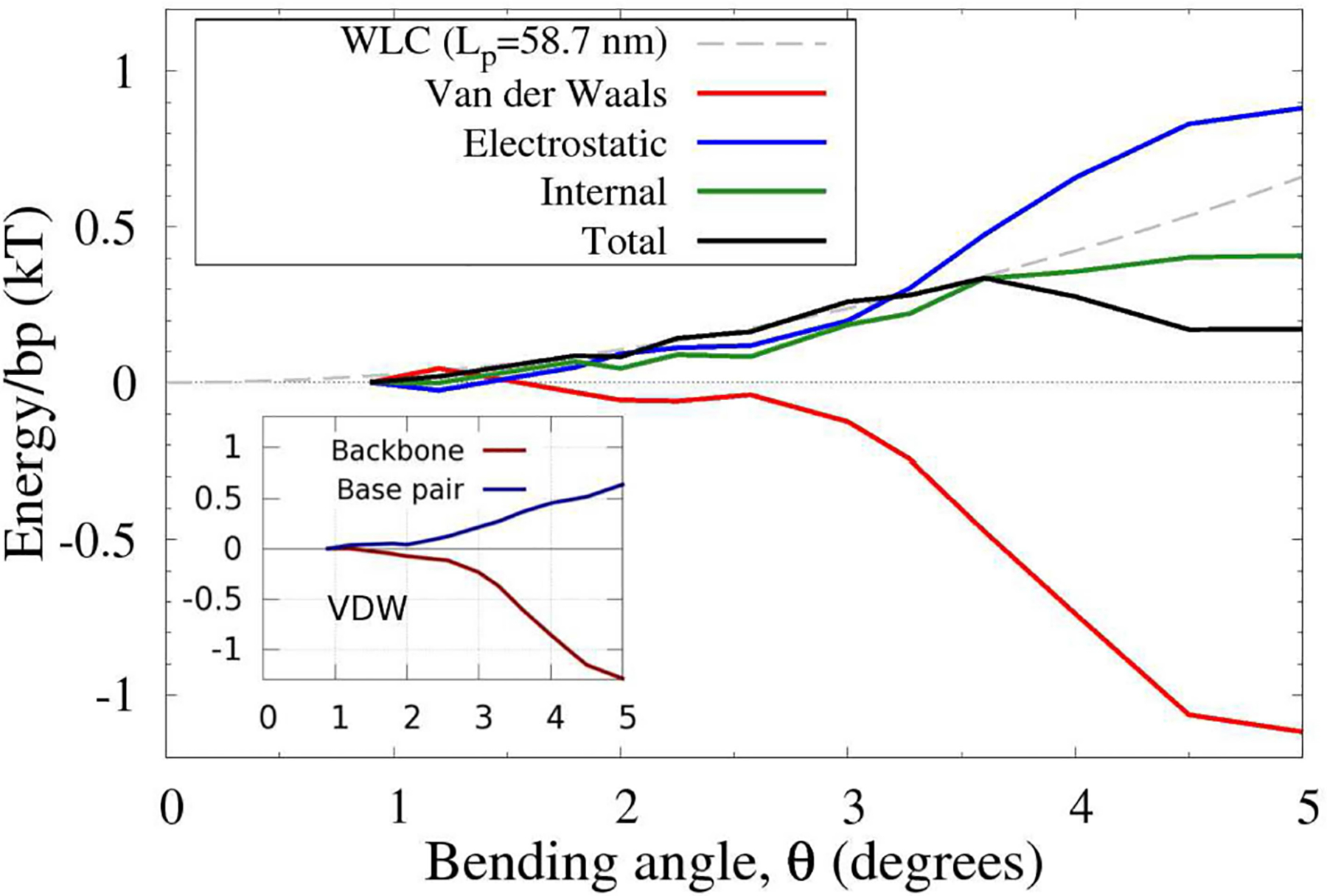
Physical components of the (total) effective DNA bending energy from Figure 6. The original range of bend angles is reduced for clarity. The energies are per base pair, inferred from all-atom MD simulations of uniformly bent DNA circles of variable lengths (50–400 bp). The main contribution to the non-convexity of the bending energy comes from the Van der Waals (VDW) interactions. The backbone-backbone part of these interactions contribute the most to the non-convexity due to a sharp increase in the attractive energy component for 3° < *θ* < 4°, as shown in the inset. For reference, a WLC fit for the small angle bends (up to ≈ 3.5°, gray dashed line) is shown.

To investigate, qualitatively, the physical origin of the non-convexity of the DNA effective bending energy we employed all-atom Molecular Dynamic (MD) simulations of uniformly bent DNA circles of a wide range of sizes, from small to very large, corresponding to almost unbent DNA (see “Methods”). Specifically, we examined the average bending energy per base pair. The total bending energy profile obtained from these simulations is shown in Figure 6, while its breakdown into components of different physical origin is given in Figure 7. One can clearly see a prominent non-convex region, in qualitative agreement with the experiment (Figure 4). The key parameter *θ*_*a*_ ≈ 1.5° from the MD simulations, which is not all that different from the value of 2.2° inferred from the experimental data (Figure 4). Some discrepancy is likely due to sequence effects [35, 88–90], force-field issues [91, 92], or the fact that the experiment-based potential in Figure 4 may itself deviate from reality to some extent, as noted in the original publication [42]. Importantly, the use of MD-derived *θ*_*a*_ = 1.5° in Equations (5) and (6) results (see Supplementary Material), in virtually the same close agreement with the cyclization experiment we have seen Figure 5, which is based on *θ*_*a*_ = 2.2° derived from experiment. This insensitivity of the prediction of ECH to the value of its key input parameter points again to robustness of the proposed framework. Variations in the DNA sequence may alter the range of bending angles over which the convex hull exists; however, a recent study [35] suggests that changing the sequence from the uniform one used here to the one employed in experiments of reference [19] has an effect of only about a factor of four on the *j-factor*, while not affecting the over-all shape of the *j-factor* dependence on the DNA loop size.

Regarding the physical origin of the non-convex effective bending energy of double-stranded DNA, the following qualitative conclusions can be made from the MD-based analysis of the DNA bending (Figure 7). For small bending angles, the total energy is reasonably well-approximated by a quadratic function. However, once the bending reaches the transition angle *θ*_*a*_, the VDW energy decreases at a rate faster than the increase of the other terms combined (Figure 7), which results in a non-convex region of the total energy, Figure 6. It is this sharp decrease in the VDW contribution that gives rise to the existence of a non-convex region in the DNA bending energy. Further analysis (inset in Figure 7), reveals that it is the attractive component of the VDW interactions between DNA backbone atoms (backbone-backbone), rather than base stacking, that is critical to the counter-intuitive sharp decrease in the total bending energy (see Supplementary Material for further atomistic details). The key role of the backbone-backbone VDW term suggests that it is the overall structure of DNA, rather than sequence details, that is responsible for the existence of the convex hull in the polymer’s effective bending energy. Further analysis is needed to reveal which specific aspects of the DNA structure are necessary and sufficient for the existence of a non-convex region in the effective bending energy. In this respect, we can infer from recent modeling studies [25, 93] that asymmetry of the DNA with respect to bending toward major vs. minor groove can lead to non-convex effective bending energy.

It is worth mentioning that local “bubbles” of broken WC bonds do not occur in our atomistic MD simulations of DNA circles *in which* the uniform bending is *deliberately* enforced by constraints on the phosphorous atoms (see “Methods”). Yet, these simulations yield a non-convex profile of the DNA bending energy (Figure 6), which, as we have demonstrated, always leads to the existence of linear “soft” bending regime. Thus, the simulations suggest that local DNA melting *may not be necessary* to explain the high flexibility of strongly bent DNA and the stark deviation of experimental *j-factors* from WLC predictions. We stress that we do not rule out “bubbles” of broken WC bonds in actual sharply bent DNA [24, 69]; but we predict that if WC bond breaking were suppressed experimentally, the *qualitative* picture of sharply bent DNA being softer than weakly bent would still hold, albeit less pronounced. The experimental *j-factors* would still deviate from WLC in a way qualitatively similar to what is currently observed in experiment (Figure 5). In particular, we predict that measured *j-factor* would still reach a minimum for small circles. An analogy can be made here with the physics behind the DNA overstretching plateau [94, 95], where the polymer extension occurs at constant force, and the stretching energy grows linearly with the polymer extension. This peculiar regime can be explained [21] via the same main argument used in the current work—the existence of a non-convex region in the polymer deformation energy. In the case of DNA overstretching, experiments have demonstrated convincingly [96] that WC bond breaking is not required for the existence of the characteristic plateau on the force-extension diagram.

At the same time, quantitative details could be different if WC bond breaking were suppressed. Note that the effective loop bending energy of ECH theory in Figure 5 comes from a statistical analysis of real protein-DNA complexes. Consequently, the ECH effective energy with parameters used in that figure, *θ*_*a*_ = 2.2° and *θ*_*b*_ = 35.8°, implicitly accounts for broken WC bonds, and other effects [33], if these occur in the DNA of the complexes. Note that these effects have been suppressed in all-atom MD simulations leading to Figure 6, which, along with inevitable force-field deficiencies, may explain why *θ*_*b*_ value that could be inferred from Figure 6 would be different from the experiment-based one from Figure 4. Fortunately, as long as *θ*_*b*_ is large enough, it has no effect on ECH predictions with respect to *j-factor*, which further supports the notion that ECH is rather robust to details of the bending potential.

### 3.6. Beyond Closed Loops: A Protein-DNA “Complex”

**FIGURE 8 |:**
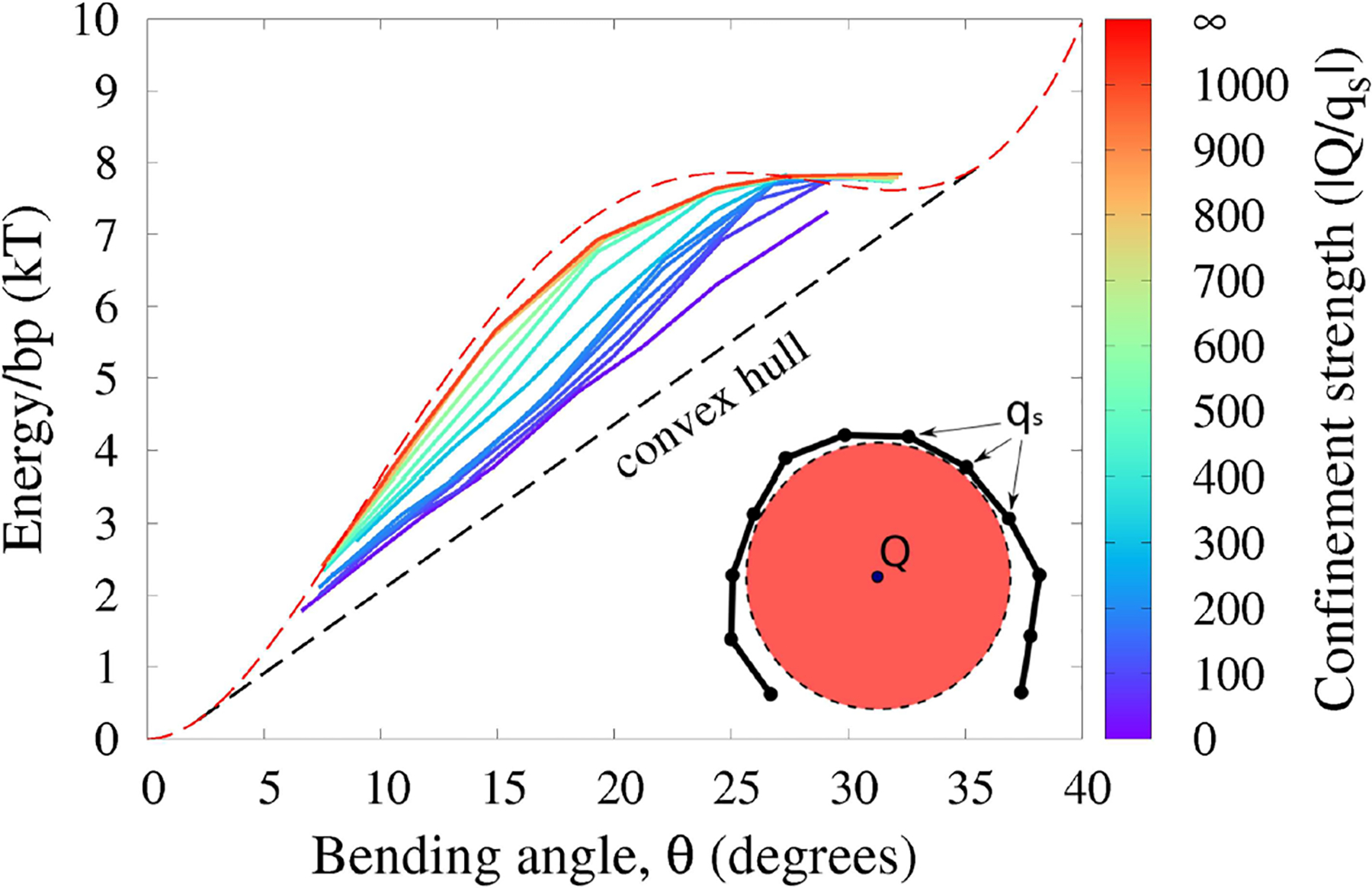
Polymer bending in a “protein-DNA complex” model with variable strength of polymer confinement and curvature (see “Methods”). The red circle represents the cylindrical charged core of the “protein” to which the oppositely charged “DNA” (black chain) is attracted. Under weak confinement, the system follows the convex hull of the effective *E*(*θ*), while approaching *E*(*θ*) (red dashed line) itself for strong confinement. Shown is the average energy per bead against the average bending angle *θ*, at different confinement strengths governed by the ratio |*Q*/*q*_*s*_| of the confining charge *Q* to the opposite charge *q*_*s*_ of the confined polymer. The intrinsic bending of the polymer is described by (experimental) *E*(*θ*) from Figure 4.

The proposed framework is based on one main assumption: despite constraints, the polymer chain is still free to explore sufficient conformational space to search for minimum energy. So far, we focused on DNA loops because of direct connection to key cyclization experiments; the single constraint $\sum_{i}^{N}\theta_{i}=\alpha =2\pi$ is minimally restrictive. However, other realistic scenarios of DNA bending, notably in protein-DNA complexes, may involve different types of constraints that can confine the polymer strongly enough to potentially violate the main assumption to various degrees. Here we investigate to which extent our main conclusion—deformation energy of strongly bent DNA follows the convex hull of *E*(*θ*)—may still hold in a model of protein-DNA complex (Figure 8 and “Methods” for details). As an example of a protein-DNA complex we choose the nucleosome [12], the fundamental repeating unit of chromatin compaction in eukaryotes, which we model here at two levels of coarse-graining. Briefly, a negatively charged polymer (the “DNA,” monomer charge *q*_*s*_) is is allowed to wrap around a charged disk (the “nucleosome core,” charge *Q*); the “core” is made impenetrable to the “DNA.” Further, the “DNA” is not allowed to slide off the “core” sideways, which is achieved through an application of appropriate positional restraints. In the simulation we vary the total positive charge *Q* of the cylindrical “nucleosome core” to modulate the electrostatic attraction of the negatively charged polymer to the “core,” which in turn modulates the degree of the polymer confinement. In the limit of very strong confinement (|*Q*/*q*_*s*_| → ∞), the polymer is forced to be confined to a circular, uniformly bent path on the surface of the cylindrical core, and has very few degrees of freedom left to explore in this regime, solid red line in Figure 8. The average bending energy in this case follows the given functional form of *E*(*θ*) (red dashed line in Figure 8), and ECH clearly does not apply. As we decrease the confinement strength, the polymer is allowed to assume non-uniform bending conformations while lowering its total bending energy. The effective bending energy per monomer begins to approach the convex hull (solid green and blue lines), making ECH more applicable. In the case of the weakest confinement (solid purple line), the polymer is still loosely bound to the core, but is allowed to relax almost completely. This is the limiting case described by our ECH model: the resulting energy per monomer follows the convex hull fairly closely.

We argue that it is this low confinement regime, where ECH is relevant, that describes the real nucleosome [13]. To substantiate the connection to the nucleosome, we provide a qualitative analysis of the corresponding energetics of the DNA binding. To this end, we model a “variable confinement nucleosome” by a coarse-grained 147-bp DNA fragment placed next to a cylinder with relative dimensions of the actual histone core [79] (see Methods); as the core charge *Q* is increased, the whole fragment starts to wrap around the cylinder once the confinement strength is |*Q*/*q*_*s*_| ~ 90. At this value of the DNA confinement, the energy cost of pulling away a fragment of ~ 20 bp in our model is ≈ 10*k*_*B*_*T*, which is qualitatively comparable to ≈ 6*k*_*B*_*T* estimated from experiment [2] as the energy needed to pull away a DNA fragment of the same length in the case of actual nucleosome. Moreover, even for higher degrees of confinement, up to |*Q*/*q*_*s*_| ≃ 200 (≃ 20*k*_*B*_*T* to pull away a 20 bp fragment), the corresponding blue lines in Figure 8 still approximate the convex hull, and so ECH is still likely applicable, at least qualitatively. Needless to say, applicability of ECH to protein-DNA complexes will require further rigorous analysis.

## 4. CONCLUSION

It is now well-established that slightly bent DNA behaves like an elastic rod—the deformation energy is a quadratic (harmonic) function of the deformation. However, recent experiments demonstrated that strong bending of small DNA fragments could no longer be described within this classical model.

Here we have proposed a novel framework for bending of polymers, which is based on the consideration of convex properties of the effective bending energy between successive monomers. Within the framework, the bending energy is harmonic for small bends, but once the average deformation reaches the convex hull of the effective bending energy function, a “phase transition” to the strongly bent regime occurs, in which the system’s energy is a linear function of the average bending angle. In this regime, which persists for as long as the average deformation is within the convex hull interval, the two states of bending co-exits: some segments are bent weakly, while others are bent strongly, with the proportion of the latter increasing with the increased average bend (e.g., shorter loops). The transition point from the harmonic to the linear bending regime occurs at the beginning of the convex hull segment—this point plays a special role in the new theory. These general considerations are expected to hold for any polymer with an effective bending energy that has a distinct non-convex region, regardless of its origin.

For generic “sequence-averaged” double-stranded DNA considered here, we conclude that the effective bending deformation energy becomes non-convex for strong bends greater than ~ 2°/bp, which corresponds to circular loops shorter than ~ 160 bp. The conclusion about the DNA bending energy being non-convex relies on an analysis of a large number of experimental protein-DNA complexes, and is consistent with the shape of the bending energy inferred from atomistic MD simulations. The simulations also yield a qualitatively similar value for the bend angle that marks the onset of the linear bending regime. Further, atomistic simulations of DNA circles reveal that attractive short-range Lennard-Jones interactions between the backbone atoms can explain the underlying non-convexity of the DNA effective bending energy, leading to the linear bending regime. We use MD simulations only for general reasoning, which is robust to details of the simulation protocol.

In this work our focus is the main principle; future refinements of the ECH theory may be able to account for details not considered here, such as sequence dependence of the DNA bending energy, the influence of torsional stress and supercoiling, etc. We have also just barely touched upon structural consequences of ECH, such as the number and distribution of “kinks” in tightly bent DNA. A detailed analysis of these features will likely lead to additional experimentally verifiable predictions of the theory. Likewise, we have derived specific mathematical expressions for bending under only one type of constraint; other relevant types of constraints need to be considered in more detail to complete the theory. Based on our analysis, the key conceptual features of ECH will likely hold.

The new theory does not contradict the conceptual basis of the classical models of DNA bending, such as WLC, but also agrees with recent experimental cyclization data on strongly bent small DNA circles [19]. A completely counter-intuitive prediction that cyclization probability reaches a minimum for very small loops has proved to be *qualitatively* consistent with additional experimental data points, not available to us when we made the prediction. We believe that the novel general framework can be used to analyze, at least conceptually, many other scenarios of strong polymer bending, and should help interpret future experimental observations.

## Supplementary Material

supplementary material

## Figures and Tables

**TABLE 1 |:** *j-factor* ratios, *J*(*L*_1_)/*J*(*L*_2_), predicted using ECH and WLC models compared with experiment [19].

L_1_(bp)	L_2_(bp)	Experiment	ECH	WLC
40	50	1.50	0.993	1.12×10^−6^
71	101	1.51×10^−1^	2.01×10^−1^	3.08×10^−6^
80	101	2.17×10^−1^	3.28×10^−1^	6.10×10^−4^
90	101	3.56×10^−1^	5.59×10^−1^	3.73×10^−2^
