# Supplementary material for: Strongly Bent Double-Stranded DNA: Reconciling Theory and Experiment
Source: Front Phys. Author manuscript; Available in PMC 2020 Jun 29. (PMC7323118; doi:10.3389/fphy.2019.00195)
Supplement: supplementary material [file NIHMS1067792-supplement-supplementary_material.pdf]

Supplementary Material to:
Strongly bent double-stranded DNA: reconciling theory and experiment

1 CONCAVE VS. CONVEX CLOSED LOOPS.

The exterior angle sum theorem is valid for convex polygons, directly applicable to the sum of the tangent vectors of a convex closed curve. We do not consider non-convex (concave) closed curves here because these can not minimize the deformation energy of a closed, linked, inextensible and uniform elastic loop, at least as long as the effective bending energy $E(\theta)$ is a monotonic function of θ, or curvature κ. (The existence of a non-convex region in $E(\theta)$ does not imply a non-monotonic $E(\theta)$.)

The outline of an intuitive proof idea is as follows. Any concave curve can be transformed – "banged out" – to eliminate a local non-convex region while bringing the bending energy down in the process, see Fig. S1 for an illustration of the process for a shallow "dimple". For a deeper "dimple", the procedure may involve two steps. First, the entire convex portion of the curve is "stretched out" via a uniform re-scaling of its curvature $\kappa(s) \to \lambda\kappa(s), 0 < \lambda < 1$. The bending energy of the convex portion of the curve will become lower during this step. Since the curve is inextensible, and there are no breaks, points A and B will move further apart as a result, thus also reducing the curvature of the non-convex "dimple" that spans the $|AB|$ segment, and hence lowering its bending energy. After that, the step in Fig. S1 is applied, making the resulting curve convex, and lowering its bending energy further. We do not pursue a more formal proof based on variational calculus.

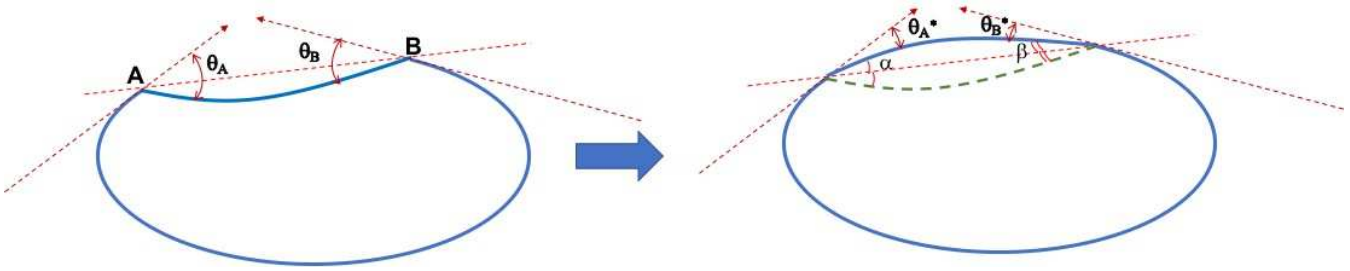

Figure S1. The bending energy of the concave closed curve (left) can be reduced by reflecting the concave portion of the curve across the convex hull line ($|AB|$, red), to produce a fully convex curve (right). The procedure reduces the bend angles θ_A and θ_B to, respectively, $\theta_A^* = \theta_A - 2\alpha$ and $\theta_B^* = \theta_B - 2\beta$, while keeping the curvature unchanged everywhere else along the curve. For a monotonic $E(\theta)$ the net result is a lower bending energy of the loop.

2 J-FACTOR ENVELOPE FUNCTIONS AND FITTING TO EXPERIMENT

The difference between ECH and WLC models is encoded in the *j-factor* via

$$j(L) \simeq \frac{k}{L_p^3}\left(\frac{L_p}{L}\right)^5 \exp\left(-\frac{\mathcal{E}_{loop}(L)}{k_BT} + \frac{L}{4L_p}\right) \quad \text{(S1)}$$

where $\mathcal{E}_{loop}$ is the bending energy of forming a loop within each model, see Main Text. For quantitative comparison with experiment we follow the standard approach and augment the above formula with an oscillatory term that accounts for periodic variations in $J(L)$ due to torsional rigidity.

Specifically, the torsional dependence of the *j-factor* is represented by a cosine function, similar to that of the original work (Shimada and Yamakawa, 1984), to oscillate between the upper and lower envelope functions, with a period of $h = 10$ bp, see Fig. 5 in the Main Text.

$$J(L) = \frac{1}{2}\Big[J_{top}\Big(1 + \cos\Big(\frac{2\pi L}{10}\Big)\Big) + J_{bot}\Big(1 - \cos\Big(\frac{2\pi L}{10}\Big)\Big)\Big] \tag{S2}$$

where the "top"/"bottom" envelope curves have the functional form of the *j-factor* in Eq. S1,

$$J_{top/bot}(L) = \frac{k_{top/bot}}{L_p^3}\left(\frac{L_p}{L}\right)^5 e^{\left(L_p\theta_a\left(2\pi - \frac{1}{2}L\theta_a\right) + \frac{L}{4L_p}\right)} \tag{S3}$$

Here we are only interested in loops of length $L < 160$ bp, since for $L > 160$ bp ECH = WLC by construction for DNA. Thus, the curve representing ECH in Fig. 5 of the main text is Eq. S2, with the corresponding top/bottom envelope functions given by Eqs. S3.

For the case of WLC we replace $\mathcal{E}_{loop}(L)$ with the appropriate expression, so $J_{top/bot}$ used in Eq. S2 are now given by:

$$J_{top/bot}(L) = \frac{k_{top/bot}}{L_p^3}\left(\frac{L_p}{L}\right)^5 e^{\left(2\pi^2\frac{L_p}{L} + \frac{L}{4L_p}\right)} \tag{S4}$$

The values of k_{top}, k_{bot} in the above equations are inferred from fitting Eq. S2 to experimental $J(L)$ points; since we have added an oscillatory part to $j(L)$ of Eq. S1, we need two parameters in the functional form of $J(L)$. For the fit we have chosen two experimental data points for loop lengths $L = 101$ bp (the *top* envelope of $j(L)$) and 106 bp (the *bottom* envelope). The reason we chose $J(L)$ that correspond to the largest L available is because the resulting fit presents the most stringent test for the model in the tight bending regime where L is small. Note that each curve in Fig. 5 of the Main Text, and Fig. S2, has its own set of best fits values of k_{top}, k_{bot}, which explains why the WLC and ECH curves do not coincide in the limit of large loops, where ECH approaches WLC.

3 ROBUSTNESS TO MODEL DETAILS

The key parameter of the ECH theory is the transition point, θ_a from the quadratic to the linear bending regime. As seen from Fig. S2, a good agreement with the experimental *j-factors* is achieved over a relatively broad range of θ_a values. Specifically, both $\theta_a = 2.2°$ inferred from experiment, and $\theta_a = 1.5°$ obtained from atomistic MD simulations (see Main Text) yield nearly the same agreement with experiment. In both cases, the counter-intuitive prediction of increased cyclization probability for very short loops hold.

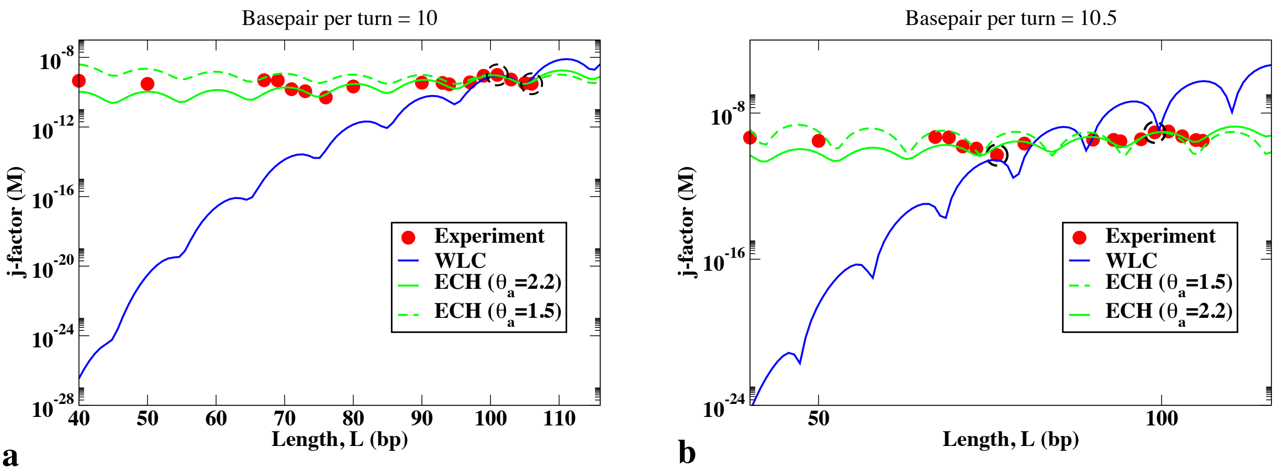

Figure S2. Robustness of ECH model predictions (green curves) to its input parameters. *j-factors* are estimated using base-pair per helical repeat $h = 10$ in (a), and $h = 10.5$ in (b). In each panel, the ECH predictions are based on two different values of the key input parameter. Solid green lines correspond to $\theta_a = 2.2°$ inferred from experiment, and dashed green lines correspond to $\theta_a = 1.5°$ from MD simulations. The fitting procedure is the same as described above, the two experimental points used to obtain the asymptotes are indicated by dashed black circles. For (a) these points are the same as in the main text.

Predictions of ECH framework are also robust to the precise value of the helical repeat parameter h used in Eqs. S2 and S1 to account for the torsional stress created by loops with non-integer number of helical repeats. The use of $h = 10.5$, more appropriate for DNA in solution(Wang, 1979), vs. $h = 10$ corresponding to classical B-form, has little affect on the agreement of the predicted *j-factors* with the experiment.

4 ORIGIN OF THE NON-CONVEX BENDING AT THE ATOMIC LEVEL

To illustrate, at the atomic level, the origin of the non-convex behavior of the backbone-backbone VDW energy, consider pairs of oxygen (O) atoms in the backbone. We choose these atoms because they have one of the lowest VDW energy minima out of all atom pairs in the DNA, and have most atom pairs within the effective short range of the interaction. The O-O VDW energy as a function of pairwise distance r_{ij} has a potential well at $r_{min} \sim 3.3$Å, Fig. S3. Further analysis of pairwise inter-atomic distances reveals that as the double helix bends, the geometry of the backbone deforms in a way that more oxygen atoms pairs fall into this well ($\sim 3.1 - 3.5$Å): beyond θ_a the accumulation of the attractive contributions begins to sharply lower the total interaction energy compared to the native, unbent state where all the oxygen atoms are significantly further apart (>4.0Å) than the well minimum. As the helix bends further, these O-O pairs eventually pass the LJ minimum, and begin to climb onto the repulsive wall, which increases the total VDW interaction energy, as seen in the corresponding figure of the main text.

REFERENCES

Shimada J, Yamakawa H. Ring-closure probabilities for twisted wormlike chains. Application to DNA. *Macromolecules* **698** (1984) 689–698.

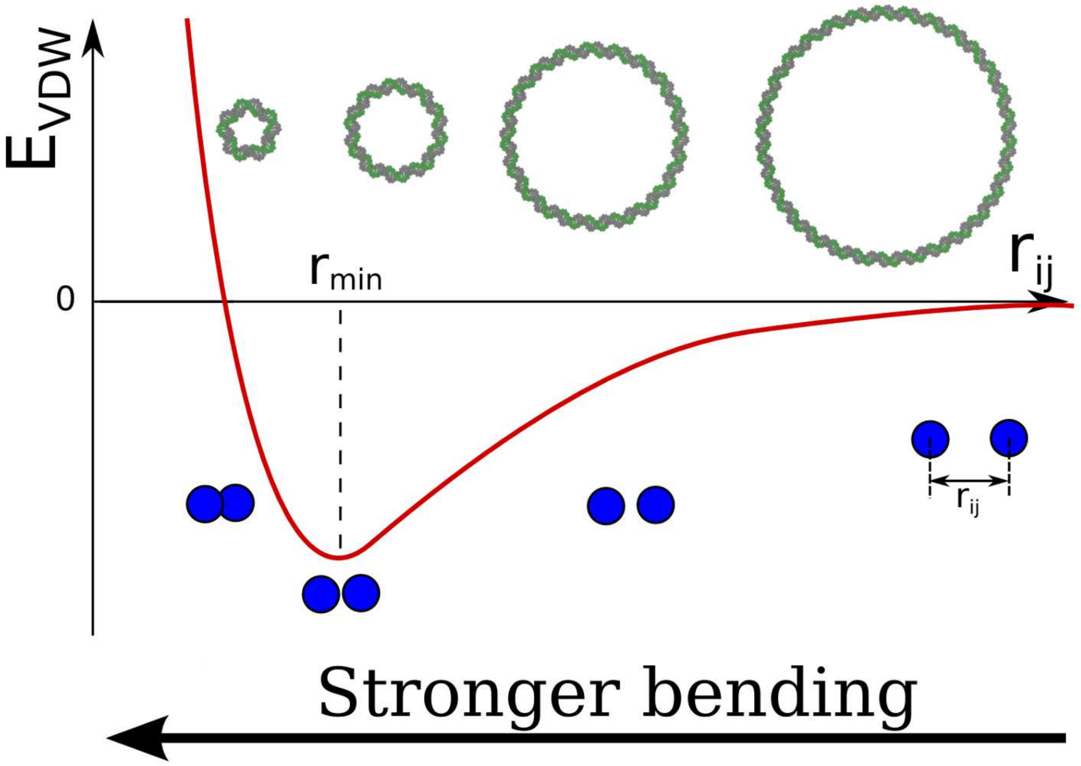

Figure S3. The increase of the bending angle of the DNA duplex causes a decrease in the average distance between atom pairs that contribute significantly to the total VDW E_{VDW}energy, modeled here as the Lennard-Jones (LJ) potential; the atoms move deeper into the LJ potential well. The shape of the well is conducive of a sharp decrease in the total VDW energy upon small changes in the atom-atom distance r_{ij} caused by the DNA bending. Once the average distance passes the LJ well minimum, the VDW energy begins to increase again.

Wang JC. Helical repeat of DNA in solution. *Proceedings of the National Academy of Sciences* **76** (1979) 200–203. doi:10.1073/pnas.76.1.200.
